# Attitudes towards Exercise, Leisure Activities, and Sedentary Behavior among Adults: A Cross-Sectional, Community-Based Study in Saudi Arabia

**DOI:** 10.3390/medicina59091524

**Published:** 2023-08-23

**Authors:** Adel Bashatah, Wajid Syed Ali, Mahmood Basil A. Al-Rawi

**Affiliations:** 1 Department of Nursing Administration and Education, College of Nursing, King Saud University, Riyadh 11451, Saudi Arabia; abashatah@ksu.edu.sa; 2 Department of Clinical Pharmacy, College of Pharmacy, King Saud University, Riyadh 11451, Saudi Arabia; 3 Department of Optometry, College of Applied Medical Sciences, King Saud University, Riyadh 11451, Saudi Arabia; basilalravi@gmail.com

**Keywords:** sedentary behavior, work-related activity, social media, leisure time, physical inactivity, Saudi Arabia

## Abstract

*Background*: Sedentary behavior has received increased attention as a threat to public health all around the world. A global effort has been made to avoid the spread of noncommunicable diseases (NCDs) that are associated with poor lifestyle practices, which rely on public awareness. As a result, the purpose of this study was to analyze the attitudes toward exercise, leisure activities, and sedentary behaviour among adults in Saudi Arabia. *Methods*: A cross-sectional study was conducted among individuals living in the Riyadh Region in Saudi Arabia. The questionnaire (26 items) used in this study was divided into four sections, and the first section comprised demographic and basic information of the respondents (6 items). The second section asked the respondents about the time spent exercising and sedentary time spent (6 items), the third section of the study comprised eight questionnaires about the frequency of sedentary activity performed during their leisure time, and the last section was about the attitude towards sedentary behavior (6 items). Descriptive and analytical statistics were done to describe the study findings. Data were analyzed using SPSS version 27. *Results*: The current findings revealed that 44% (*n* = 305) of the respondents performed exercise 1–2 days a week, and 16.7% (*n* = 116) never performed any exercise. Furthermore, a considerable percentage of the respondents spent >4 h in a day as sedentary. Most of the sedentary time was spent on work relating activities 62% (*n* = 430), followed by time spent on coffee 36.4% (*n* = 252), business relating activity 22.5% (*n* = 156), and social media 8.9% (*n* = 62). In this study, most of the respondents agreed that sitting for a prolonged time might negatively impact their health. Most of the respondents showed positive attitudes towards sedentary behavior. Males were statistically more likely than females to exercise 1–2 days per week (*p* < 0.001). Being male and being married were both significantly associated with sedentary behavior (*p* < 0.001). In addition, there was a significant association between participants’ sleeping status and physical activity per week, where those who slept 5–6 h often performed physical activity, indicating a significant difference (*p* < 0.001) than respondents who slept 7–8 or >8 h. The participant’s age was also found to have a significant association with engaging in physical exercise (*p* < 0.001). *Conclusions*: The results of this study showed that Saudi adults are highly sedentary and inactive, though knowing the harmful consequences of inactivity. Therefore, a national active living policy must be adopted to discourage inactivity and being sedentary and encourage active living in Saudi Arabia.

## 1. Introduction

Sedentary behavior (SB) is defined as engaging in sociocultural activities that reduce human energy expenditure by 1.5 metabolic equivalents (METs) above resting [1,2]. Sedentary behavior is often associated with behaviors such as sleeping, sitting for a long time, commuting, in the workplace and at home, and lying down [1,2]. Furthermore, over the last few years, the time spent watching television and time spent on the internet has increased dramatically [3]. Modern civilizations have created harmful behaviors that are increasingly concerning, ranging from watching television (TV) on the couch to performing professions that do not need any physical activity [3]. Furthermore, utilizing the advantage of technology and urbanization has resulted in a sedentary lifestyle among individuals worldwide [4].

Sedentary behavior is the most important yet under-addressed public health challenge [5,6]. It was estimated that around 60 to 85% of people worldwide are suffering from sedentary behavior, resulting in the development of various chronic lifestyle-related diseases in both developed and developing countries worldwide [6]. Sedentary behavior is associated with multiple negative consequences, including an increased risk of cardiovascular and metabolic disorders, musculoskeletal disorders such as arthralgia, osteoporosis, anxiety, depression, and those who are vulnerable to mobility. Furthermore, sedentary people are more likely to develop obesity in comparison to active people [7,8,9,10,11]. The World Health Organization (WHO) reports sedentary behavior as the top 10 causes of death and disability in the world [5]. Around 3.2 million deaths per year are reportedly due to sedentary behavior [8,9]. A third of the people in the world who are 15 years old and older have insufficient physical activity, which hurts their health. It was found that the daily average sedentary behavior among individuals was 7–8 h [10]. Prolonged periods of inactivity can slow down the metabolism and make it harder for the body to maintain healthy blood sugar levels, blood pressure, and fat-burning processes, weakening the immune system and speeding up the aging process [10,11,12].

A more recent study about the fitness trends around the globe revealed that wearable technology, strength training with free weights, and outdoor activities remain within the top five fitness trends in the USA, while, in Spain, exercise is considered a medicine and hit the top 20 trends for the first time in 2023 [13]. However, this trend has remained a top 20 trend in other worldwide surveys since 2017. In Australia fitness programs for older adults, fitness training continues to be very popular and stands for first and second on the global trend list, while strength training with free weights has remained a hot trend for the past 3 years [13]. On the other hand, another recent study in Europe revealed personal training, high-intensity interval training, body weight training, functional fitness, and small group personal training were identified as the top five trends, respectively [14].

According to WHO estimates, about 500 million new cases of preventable noncommunicable diseases (NCDs) will be diagnosed worldwide by 2030. To improve people’s quality of life, the WHO launched a remarkable new concept known as the Global Action Plan on Physical Activity 2018–2030 (GAPPA) [15]. As part of the GAPA recommendations, countries are urged to develop and implement dedicated comprehensive national policies to ensure more secure and safer roads for cycling and walking to increase levels of physical activity within their populations, so the GAPPA’s main goal is to pursue a 15% reduction in the prevalence of physical inactivity worldwide by 2030 [15,16]. It is worth noting that people’s levels of inactivity vary greatly throughout countries, reaching as high as 80% in some adult subgroups [15,16]. Adults in the Eastern Mediterranean Region, Americas, Europe, and Western Pacific Regions have reported high levels of physical inactivity, whereas adults in Southeast Asia are found to have lower levels of physical inactivity [15]. Saudi Arabia and the neighboring Gulf Cooperation Council countries have witnessed substantial lifestyle changes as a result of rapid urbanization, socioeconomic development, and a shift toward sedentary behavior [17,18]. On the other hand, sedentary time has been increasing among individuals in Saudi Arabia and other countries [19,20,21]. For instance, a prior study found that the average amount of sitting time was 690 min per day on workdays and 575 min per day on weekends [19]. The degree of education, the number of children, and employment in the private sector were all predictors of the workday sitting time [19]. The presence of children, being single, and living in a small house were revealed to be predictive factors for non-workday sitting time [19]. On the other hand, it was found that the majority of Canadians spent time being sedentary [21].

The physical demands of daily life have been greatly lessened as a result of lifestyle changes, which have also encouraged sedentary behavior in both young and old people. Saudi Arabia (KSA) is a country where physical inactivity is reportedly high [22,23]. On the other hand, the literature has revealed that a sizable proportion of the Saudi population does not reach the World Health Organization (WHO) requirements for physical activity guidelines [20,23,24,25,26]. According to a recent in Saudi Arabia, the prevalence of physical inactivity (PIA) was 71.70% among adult males and 91.10% among females [22]. Similarly, in another recent study among young adults, the prevalence of physical inactivity was 37% [27]. In a population-based study of Saudi adults, Al-Nozha et al. discovered that 96.1% of them were physically inactive, showing a fairly high prevalence of inactivity [28]. Another finding suggested that 80% of Saudi adults are not achieving adequate levels of PA with beneficial health effects [20]. One of the key recognized hurdles to high sedentary behavior and physical inactivity was a lack of time and facilities [20,22,26]. Despite the rising prevalence of physical inactivity, which considerably influence the rates of morbidity and mortality, there have only been a few studies that investigated sedentary behavior and its related factors among people living in Riyadh, Saudi Arabia, from a community viewpoint. Additionally, it is hypothesized that, since sedentary behavior and inactivity are becoming more common among people, especially older adults and women, raising awareness of the dangers of sedentary behavior and changing people’s attitudes about it could help maintain their overall health. Therefore, the purpose of this study was to analyze the attitudes toward exercise, leisure activities, and sedentary behaviour among adults in Saudi Arabia.

## 2. Methods

### 2.1. Study Design, Settings, and Population

Between April 2023 and June 2023, a cross-sectional study was carried out in Saudi Arabia using online self-reporting questionnaires. Adults living in the Riyadh Region of Saudi Arabia who are Saudi nationals, over the age of 18, able to give informed consent, and who agree to engage in the study were included in this investigation. This study was conducted according to the guidelines of the human research Declaration of Helsinki’s standards. Before beginning the study, informed consent was obtained from all Saudi adults by utilizing online survey technologies after a brief description of the goals and advantages was given, with an emphasis on the confidentiality of the study’s data and its use solely for scientific research. Furthermore, participation was voluntary, no monetary or non-monetary rewards were provided, and respondents were free to leave at any time during the study period.

Riyadh, the capital of the Kingdom of Saudi Arabia, is the most populous city in the nation and has a total area of 1798 square kilometers [29]. It is located on a plateau in the middle of the Arabian Peninsula. Since the fourteenth century, the city has expanded and has gone through numerous changes due to its rapid economic changes [29]. Furthermore, it is estimated that a total of 4300 people live in each square kilometer, which is the population density [29]. The most recent estimates place Riyadh’s population at 7.68 million, making up 24.9% of Saudi Arabia’s overall population and consisting of 55% men and 45% women [29]. In this study, we defined physical activity as a type of activity related to any kind of work, athletic, domestic, and other activities. Exercise is a type of physical activity that is planned, scheduled, and repeated for the enhancement or maintenance of physical fitness as a primary or secondary goal [30,31,32]. On the other hand, sedentary behavior is time spent sitting or lying down. This term derives from the Latin word sedere, which means to sit [33]. Furthermore, family time is defined as spending quality time sitting with their family watching television, eating, and sitting with various types of communication between family; kids; and their parents, grandparents, and estranged siblings [34].

### 2.2. Study Questionnaire

The questionnaires used in this study were adopted after a thorough review of the recently published literature from the World Health Organization (WHO), the Centers for Disease Control and Prevention (CDC), and the Saudi Ministry of Health [5,20]. A self-administered questionnaire was developed. The four sections made up the questionnaire, with a total of 26 items. The first section comprised demographic and basic information of the respondents (6 items). The second section asked the respondents about their time spent exercising, physical activity, and sedentary time (6 items), the third section of the study comprised seven questionnaires about the frequency of sedentary activity performed during their leisure time, and the last section was about their attitude towards sedentary behavior (6 items). All these questionnaires were multiple choice and on a 5-point Likert scale (attitudes items). A score of 5 was assigned for strongly agreeing, a score of 4 for agreeing, a score of 3 for neutrality, a score of 2 for disagreeing, and a score of 1 for severely disagreeing, with a rating for their physical activity over the previous week, their health condition, and their mental state.

In two steps, the designed questionnaire was validated. First, a group of research professionals in the relevant domains received the first draft. Second, pilot research involving 10 people was carried out. The final questionnaire then incorporated changes from the pilot research. By utilizing SPSS version 27 to calculate Cronbach’s alpha, a reliability test was conducted. The final analysis did not include the pilot study’s data. The finished questionnaire was then distributed to all Saudi adults utilizing online survey technologies. The data collection was followed by a convenience sampling strategy (which is a nonprobability sampling that involves the sample being drawn from that part of the population that is close at hand). For data collection, electronic questionnaires using google forms were prepared and distributed. We targeted twelve hundred adults, and we sent them the questionnaires. To achieve the number of responses, we sent a reminder and encouraged the respondents to participate and complete the study questionnaires. We received 912 filled questionnaires. Although *n* = 219 questionnaires were excluded from the study due to mismatching the inclusion criteria and incomplete answers. Therefore, we included a total of 693 respondents in the final analysis.

### 2.3. Statistical Analysis

For data analysis, IBM Statistics SPSS version 27 was used. Each item of data was scrutinized for accuracy. Descriptive and inferential statistics, such as frequency distributions (*n*), percentages (%), and means and standard deviations (Std), were used to report the findings. To find out the association between variables, a chi-square/Fisher’s exact test was used, with *p* < 0.005 indicating a statistically significant difference.

## 3. Results

### 3.1. Sociodemographic Characteristics

A total of 693 individuals aged ≥18 years living in Saudi Arabia participated in this study. More than two-thirds (67.7%; *n* = 469) of the respondents were males, and the mean age of the study respondents was 35.5 years. The majority of the respondents were Saudi nationals (609 (87.9%)). The proportion of currently married was 56% (*n* = 388), while those single were 44% (*n* = 305). Only 20.9% of individuals were medical and 19.9% were pharmacy students, and most of the respondents (*n* = 535; 77.2%) never smoked. Respondents’ demographic characteristics and professional information are summarized in Table 1.

The current findings revealed that 44% (*n* = 305) of the individuals spent 1–2 days a week exercising, and 16.7% (*n* = 116) never performed any exercise. With regards to time spent sedentary, 13.6% (*n* = 94) of them spent >4 h a day inactive, followed by 3–4 h by 25% (*n* = 173) of the individuals. With regards to physical activity per week, only 3.0% (*n* = 21) practiced it always, and 17.2% of them performed sometimes in a week, while 48.9% of them rarely performed physical activity. With regards to weekends, 16.7% of them always performed physical activity and 19.6% sometimes. Detailed information about the frequencies of exercises and spending sedentary time per week are described in Table 2.

**Sedentary time on different activities (**Table 3**)**.

**Social media:** According to this study, on a typical day, more than one-third (32.9%) of the respondents spent sedentary time on social media, followed by 24.8% of them spent one hour, 12.3% two hours, and 12.0% spent more than 30 min.

*Business-relating activity:* Just one-third of the respondents spent less than 30 min on business-related activity. In addition, 22.5% spent more than 4 h, 20.1% spent more than 2 h, and just 15.2% spent 30 min of their inactive time on business-related activity.

Family time: When it comes to spending sedentary time with family, 40.1% of respondents did so for 2 h, followed by 38.2% for 3–4 h and only 7.6% for an hour.

Watching television (TV) and playing Video games: More than one-fourth of participants watched TV for 30 min, compared to more than half (53.7%) who watched it for less than 30 min. Only 10.8% of people watched it for an hour. In this study, only 12.8% of respondents spent more than two hours playing video games during their sedentary time, and 5.3% of respondents spent more than four hours. The majority of respondents (78.2%) spent less than 30 min playing video games during their inactive time.

Work relating activities and time spent on Coffee: More than two-thirds of the respondents spent > 4 h on work-related activities, while only 15.4% of the respondents spent 3–4 h, and only 12% spent 1 h of their sedentary time on work-related activities. More than one-third of the respondents spent > 4 h having coffee in a day, while 28.6% of the respondents spent 2 h, followed by 10.2% who spent 30 min of their sedentary time having coffee.

**Figure 1:**
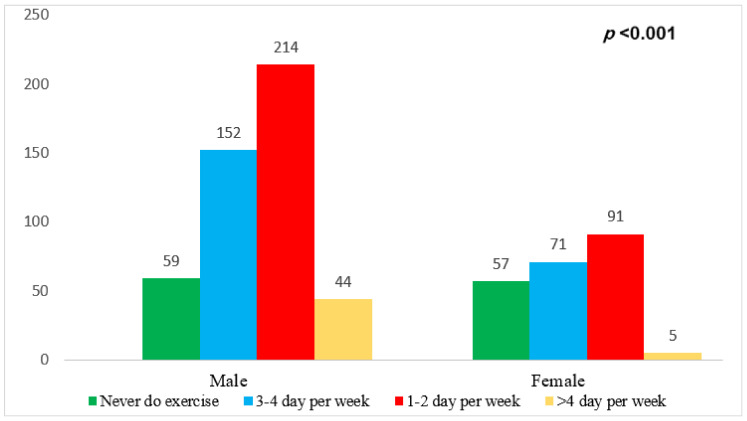
Association between exercise per week and the participants’ sex.

Figure 1 depicts the respondents’ exercise status per week based on their sex. The four colors show the levels of agreement with the phrase “exercising per week” among respondents. From left to right, the bar depicts responses with the frequency of exercise per week. A chi-square test for the relationship between sex and agreement level yielded a *p*-value *p* < 0.001, with males more likely to perform exercise 1–2 days per week compared to females, indicating a significant association.

**Figure 2:**
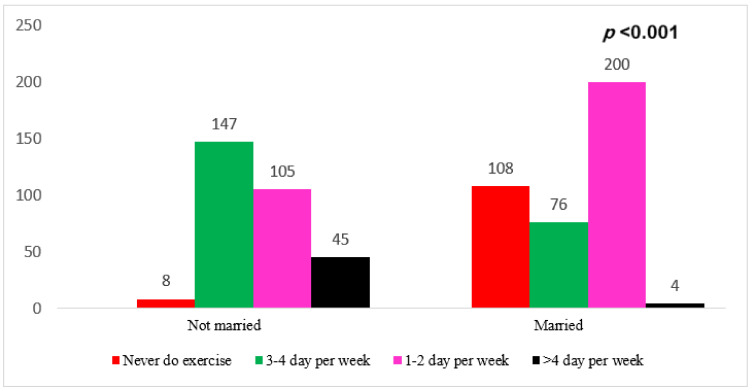
Association between exercise per week and the participants’ marital Status.

As demonstrated in Figure 2, participants who were married and exercised just 1–2 days per week had a significant association between their marital status and their response to exercise (*p* < 0.001).

**Figure 3:**
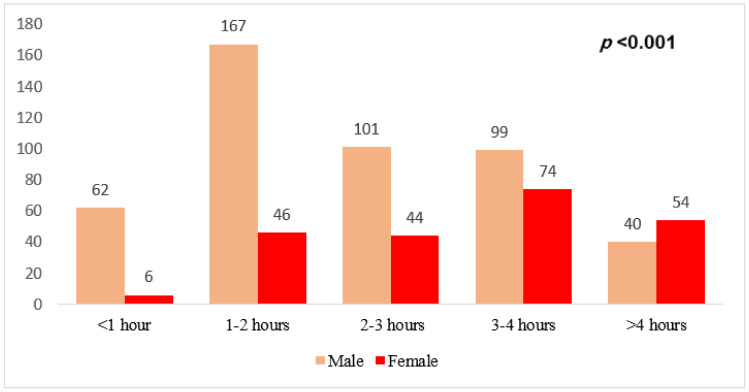
Association between sedentary time per day and the participants’ sex.

Figure 3 depicts the respondents’ sex compared to sedentary time per day. The two colors show the degrees of agreement with the phrase “time spent on sedentary per day. A chi-square test for the relationship between sex and agreement level yielded a *p*-value of <0.001, where males were more likely sedentary for 1–2 h compared to females, indicating a significant association between the status of sedentary time per day and respondents’ sex (*p* < 0.001).

**Figure 4:**
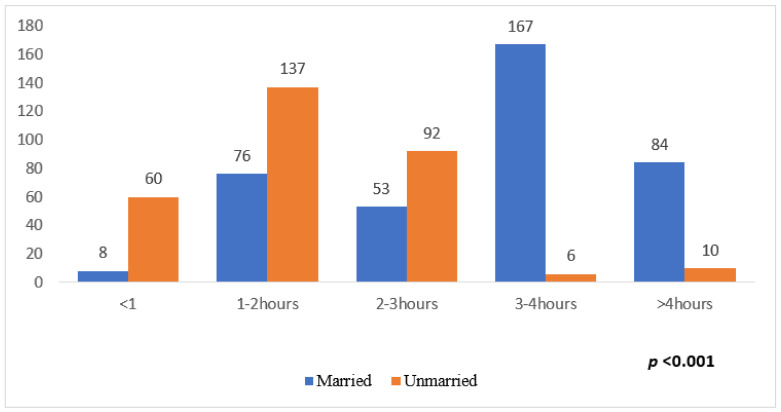
Association between sedentary time per day and the participants’ marital status.

Figure 4 depicts the participants’ marital status and sedentary time per day. A chi-square test for the relationship between marital status and agreement level yielded a *p*-value of <0.001, where those married were more likely to spend between 3 and 4 h of sedentary time in comparison to unmarried individuals (*p* < 0.001), as shown in Figure 4.

Table 4 shows an association between time spent on physical activity and the participants’ sleeping status and age. The findings of this study revealed that there was a significant association between participants’ sleeping status and time spent on physical activity per week, where those who slept 5–6 h often performed physical activity, indicating a significant difference (*p* < 0.001) compared to respondents who slept 7–8 or >8 h, as shown in Table 4. Similarly, the age of the participant was found to have a significant association with performing physical activity (*p* < 0.001).

Similarly, those who were often active physically in a week were found to have excellent health status compared to individuals who were only sometimes or rarely active in a week (*p* < 0.001) (Table 5). The findings of this study revealed that there was a significant association between professional classification and physical activity per week, where 96.2% of the students often carried out physical activity compared to the others (*p* < 0.001) (Table 6).

### 3.2. Attitudes towards Sedentary Behavior

When the respondents were asked whether sitting for a prolonged time might negatively impact their health, 80.2% agreed to this statement, while 44.9% of the participants’ opinions were neutral toward the statement that sitting for prolonged periods will not negatively impact their health if they regularly exercise during the day. A large proportion (83.1%) of the respondents agreed that sitting for prolonged periods could negatively impact their mental–emotional state. Moreover, when the study cohort was asked whether they agreed with the statement that there is a strong relationship between backpain and prolonged sitting hours, 79.3% of the respondents agreed to this statement, while 71.4% of the respondents agreed that there is a strong relationship between social media and prolonged sitting hours. Furthermore, almost 58.6% of the respondents agreed that there is a strong relationship between coffee–dessert time and prolonged sitting hours (Table 7).

## 4. Discussion

This study evaluated sedentary behavior and attitudes toward sedentary behavior among adults in Riyadh, Saudi Arabia. Not much literature was identified nationally and internationally about sedentary behavior; however, most of the literature reported on the prevalence of physical activity [17,20,24,27]. This study adds a significant contribution to the assessment of sedentary behavior among individuals in Saudi Arabia and other countries and can serve as a reference for the much-needed upcoming studies. Our study found that 44% of the individuals spent 1–2 days a week exercising. With regards to time spent as sedentary, 13.6% of them spent >4 h a day, followed by 3–4 h by 25% of the individuals, and more than one-third of respondents had 1–2 h of sedentary time. With regards to physical activity per week, only 3.0% practiced it always, 17.2% of them performed it sometimes in a week, while 48.9% of them rarely performed physical activity. During their sedentary time, 40.1% of respondents spent 2 h, and 38.2% 3–4 h, with their family, while 32.9% of them spent the time on social media. In addition to this, the majority of the respondents showed a positive attitude towards sedentary behavior and agreed that sitting for a prolonged time might negatively impact their health (80.2% agreed). These findings were comparable to a recent study in Ethiopia, where the authors discovered that the average sedentary time was 6.2 h (±2.2) on weekends and 5.8 h (±2.3) on working days [35]. Similarly, another recent study on physical activity found that physically active people were sedentary for an average of 9.55 h per day [20]. In contrast, other studies found that physically active people sat for more than 8 h per day [20,26,36,37]. A similar study among the Saudi population found a significant prevalence of sedentary behavior [37]. Sedentary habits are becoming increasingly common among adults as a result of technology improvement and social ties among the general population, resulting in increased sedentary behavior and the reduction of physical effort during leisure time [20,37]. Furthermore, persons with high levels of sedentary activity are more likely to have unfavorable health effects. Proper worksite interventions must be undertaken to improve employee health. The increased sedentary activity in the current study might be due to the cultural, socioeconomic, and lifestyle factors in the studied population.

Saudi Arabia’s standard of living has substantially increased as a result of its rapid economic expansion during such a transition period. Saudi inhabitants have seen a large rise in free time [27]. It is not surprising that having more free time leads to more sedentary behavior. Sedentary time spent performing various activities has increased significantly in recent decades [20,26,27,35]. In this study, it has been found that individuals always focused on spending more time on video games, YouTube, Facebook, Instagram, and Twitter, with family and business-related activities. Our study findings were comparable to similar the findings published by Motuma et al. in 2021 [35], where the authors reported that business-related activities or office work, watching TV/video playing, social media, and interacting with friends and/or family [35]. Similarly, another study among Canadians reported that the most common sedentary behavior was spent watching television or video viewing and time spent using computers and electronic devices. Television and video viewing appear to have increased in older adults [21]. These findings are alarming in creating awareness about the harmful events associated with sedentary behavior. Furthermore, it has been demonstrated that exercise and physical activity improve the quality of life, particularly for overweight and obese people, and help prevent a variety of metabolic diseases, emphasizing the significance of a multicomponent exercise approach to better cardiometabolic health [31].

According to a national study conducted in Armenia, the rate of physical inactivity during leisure time was 86.2% [36]. A follow-up study on adolescents in Brazil found that they spent around 9 h per day on total sedentary activities [37]. In our study, 23.2% of respondents spent 30 min on social media, one-third spent less than 30 min on a business-related activity, and 39.8% spent 3–4 h’ inactive time with family. Almost half of the respondents (50%) spent less than 30 min watching TV, and a large proportion (74%) of the respondents spent less than 30 min playing video games during their sedentary time. For instance, a study conducted on retired men and women concluded that the increase in time spent watching TV was more than twice as high in retirees as in workers (11.3 min/day for men and 10.0 min/day for women) [38]. A recent study conducted on adolescents concluded that they spent about 3.7 h a day on screentime activities [37]. A previous study on young people concluded that 2 to 4 h are usually dedicated to screentime (television, computers, video games, and smartphones) [39]. Technological advances are more readily available to working and educated adults. It is likely that youths and working adults lead a more Western lifestyle and engage in more screen-based leisure time, which may account for the higher level of sedentary behavior than the previous generation.

A novel and challenging topic for research in exercise science, behavioral science, and population health is those adults who meet the public health guidelines regarding physical activity, as their metabolic health is compromised if they sit for long periods [40]. However, in this context, 76% of the study subjects agreed that prolonged sitting might negatively impact their health. Nevertheless, it is worth noting that the current study provided a counter-case to previous studies by identifying that 48.8% of the respondents did not feel comfortable with their workstations, and 73.6% felt exhausted during the workday. Additionally, 6.3% suffered from hypertension, and 11.2% of them reported hyperlipidemia [41].

The most potential target for sedentary behavior intervention are desk workers and students, because they are more likely to engage in prolonged sitting, which has minimal physical demands. In the current findings, 75.2% of respondents agreed that there is a relationship between backpain and prolonged sitting hours. Many previous studies concluded that continuous sitting is the source of backpain [31,42,43,44,45]. For instance, a previous study among office workers concluded that 53.2% of them suffered from backpain. In addition to this, previous studies have found that sitting for over half of a workday, accompanied by awkward postures or often working in a forward bent position, increases the likelihood of backpain [42,43,44,45]. This could be explained by the fact that prolonged sitting puts an increasing amount of strain on the back, neck, arms, and legs. It also puts a lot of pressure on the back muscles and spinal discs, which is a major cause of backpain. [45] In addition to this sedentary behavior, less physical activity increases the severity of low backpain [46]. It was also found that people who spend an average of ten hours per day sitting or lying around are at a higher risk for depression [11,47,48]. The reason for this may be that, since sedentary time is typically spent alone, it stops people from interacting with one another. This leads to feelings of loneliness and negative emotions, which can have an impact on their psychological well-being. Furthermore, a European teenage survey found that long-term inactivity among individuals was associated with an increase in inflammatory markers such as IL-6, which correlate with anxiety and depression [48,49]. In addition, being sedentary, particularly at night, may alter circadian rhythms and displace sleep [48,50], which can affect the mental status of individuals [48,49,50].

It is known that sedentary and physical inactivity raises individuals’ disease status. For example, prolonged sitting and a lack of physical activity are associated with obesity, diabetes, and cardiovascular diseases, which may increase the chances of early death and aging, leading to a disturbed quality of life [20,31]. Although raising awareness of the risk factors and complications associated with sedentary behavior has been a common strategy for controlling disease incidence. An increasing prevalence of noncommunicable diseases with lifestyle-related and associated morbidity and mortality in Saudi Arabia means counteracting measures are urgently needed.

This study had several limitations. First, because the study was self-reported, recall bias cannot be completely ruled out. Second, the study was conducted in Saudi Arabia’s center region; third, there was a low participation rate, rendering it both nationally and internationally unrepresentative and, hence, not applicable internationally. Fourth, the survey only included Saudi individuals. Fifth, the study was distributed online, and only individuals with internet access and the ability to read were allowed to participate. Sixth, the results were collected using Google Forms. Lastly, the absence of a comparison group or control group, habitual physical activity, and eating habits assessments might be possible additional limitations. Despite these limitations, our study had several perks. This study began by looking at the importance of the public’s understanding in raising awareness of sedentary behavior and the benefits of physical activity in enhancing one’s overall health. Additionally, there have been a dearth of such studies in this field, particularly among the Arab population. Additionally, we concentrated on Saudi adults, because the prevalence of the majority of metabolic disorders, such as obesity and diabetes, was said to be higher among Arabs. In addition, we looked at how respondents’ attitudes toward exercise, leisure time, and sedentary behavior might improve their general health and result in better health outcomes. As a result, our findings point to the importance of educating young people about PA’s effectiveness and preparing them to advocate for a healthy lifestyle.

## 5. Conclusions

The present study found that most respondents had a favorable attitude toward sedentary behavior, and 80.2% and 80.2% of them agreed that spending a lot of time sedentary could be bad for their overall health. There is a need for the promotion of healthy lifestyles in these populations. However, in this study, 16.7% of them never performed any exercise. Furthermore, 13.6% of the respondents spent >4 h a day sedentary. Most of the sedentary time was spent on social media, followed by work-related activities, time spent on coffee, and business-related activity. Therefore, it is recommended that, to address the causes of sedentary time, individuals and students should spend some time engaging in physical activity. This can be accomplished by supportive parents and by employers providing additional support for their employees to engage in a variety of health-promoting activities through a coordinated effort at the local, national, and international levels across numerous sectors and disciplines. In addition, this information could be highly useful for policymakers to develop a national action plan for physical activity and to guide the development of national physical activity guidelines to enable health professionals to promote and encourage healthy habits for patients and communities by providing routine check-ups and free counseling on how to increase healthy activities, improve fitness, and reduce sedentary behavior, enhancing the quality of life for their patients.

## Figures and Tables

**Table 1:** Sociodemographic characteristics of the study participants.

Characters	Frequency (*n*)	Percentage (%)
Sex		
Male	469	67.7%
Female	224	32.3%
Marital status		
Married	388	56%
Not married	305	44%
Age (years)		
23–25	327	47.2%
26–29	71	10.2%
30–35	118	17%
36–39	129	18.6%
>40	48	6.9%
Profession		
University employee	66	9.5%
Business Man	20	2.9%
Student at the College of Dentistry	40	5.8%
Working in the private sector	50	7.2%
House Wife	40	5.8%
Medicine Student	145	20.9%
Working in the government sector	194	28.0%
Pharmacy Student	138	19.9%
Sleeping hours per day		
<5 h	11	1.6%
5–6 h	325	46.9%
7–8 h	341	49.2%
>8 h	16	2.3%
Smoking status		
Current smoker	90	13%
Previous smoker	68	9.8%
Never smoker	535	77.2%

**Table 2:** Time spent exercising and physical activity and sedentary time spent.

Characters	Frequency (*n*)	Percentage (%)
Exercise per week		
>4 days per week	49	7.1%
3–4 days per week	223	32.2%
1–2 days per week	305	44%
Never do exercise	116	16.7%
Sedentary time in a day		
<1 h	68	9.8%
1–2 h	213	30.7%
2–3 h	145	20.9%
3–4 h	173	25%
>4 h	94	13.6%
Physical activity level per weekday		
Never	82	11.8%
Rarely	339	48.9%
Sometimes	119	17.2%
Always	21	3.0%
Often	132	19.0%
Rate your self-regarding your physical activity level per a weekend day		
Never	52	7.5%
Rarely	340	49.1%
Sometimes	136	19.6%
Always	116	16.7%
Often	49	7.1%
Rate your health state		
Poor	46	6.6%
Fair	101	14.6%
Good	50	7.2%
Very good	181	26.1%
Excellent	315	45.5%
Rate your mental-emotional state		
Poor	8	1.2%
Fair	72	10.4%
Good	51	7.4%
Very good	433	62.5%
Excellent	129	18.6%

**Table 3:** Information about the respondents’ sedentary hours per day for each leisure activity.

Sedentary Hours per Day for Each Leisure Activity	Frequency (*n*)	Percentage (%)
Social media		
<30 min	83	12%
30 min	228	32.9%
1 h	172	24.8%
2 h	85	12.3%
3–4 h	63	9.1%
>4 h	62	8.9%
Business-related activity		
<30 min	207	29.9%
30 min	105	15.2%
1 h	82	11.8%
2 h	139	20.1%
3–4 h	4	0.6%
>4 h	156	22.5%
Family time		
<30 min	51	7.4%
30 min	7	1%
1 h	53	7.6%
2 h	278	40.1%
3–4 h	265	38.2%
>4 h	39	5.6%
Watching TV		
<30 min	372	53.7%
30 min	167	24.1%
1 h	75	10.8%
2 h	55	7.9%
3–4 h	2	0.3%
>4 h	22	3.2%
Video games		
<30 min	542	78.2%
30 min	8	1.2%
1 h	14	2.0%
2 h	89	12.8%
3–4 h	37	5.3%
>4 h	3	0.4%
Work-related activities		
<30 min	48	6.9%
30 min	8	1.2%
1 h	83	12%
2 h	17	2.5%
3–4 h	107	15.4%
>4 h	430	62%
Coffee time		
<30 min	70	10.1%
30 min	71	10.2%
1 h	58	8.4%
2 h	198	28.6%
3–4 h	44	6.3%
>4 h	252	36.4%

**Table 4:** Association between time spent on physical activity and the participants’ sleeping status and age.

Variables		Physical Activity Level per a Week					*p*-Value
		Often *n* (%)	Always *n* (%)	Sometimes *n* (%)	Rarely *n* (%)	Never *n* (%)	
Sleeping hours							
<5 h	Count	0	0	6	2	3	<0.001
	% within Sleeping hours per day	0.0%	0.0%	54.5%	18.2%	27.3%	
5–6 h	Count	90	8	32	121	74	
	% within Sleeping hours per day	27.7%	2.5%	9.8%	37.2%	22.8%	
7–8 h	Count	42	9	80	206	4	
	% within Sleeping hours per day	12.3%	2.6%	23.5%	60.4%	1.2%	
>8 h	Count	0	4	1	10	1	
	% within Sleeping hours per day	0.0%	25.0%	6.3%	62.5%	6.3%	
Age(In years)							
23–25	Count	127	0	41	158	1	<0.001
	% within Age	38.8%	0.0%	12.5%	48.3%	0.3%	
26–29	Count	1	2	30	4	34	
	% within Age	1.4%	2.8%	42.3%	5.6%	47.9%	
30–35	Count	1	3	11	64	39	
	% within Age	0.8%	2.5%	9.3%	54.2%	33.1%	
36–39	Count	0	3	23	99	4	
	% within Age	0.0%	2.3%	17.8%	76.7%	3.1%	
>40	Count	3	13	14	14	4	
	% within Age	6.3%	27.1%	29.2%	29.2%	8.3%	

**Table 5:** Association between time spent on physical activity and the participants’ health status.

Physical Activity per week		Health State					*p*-Value
		Excellent *n* (%)	Very Good *n* (%)	Good *n* (%)	Fair *n* (%)	Poor *n* (%)	
Often	Count	129	3	0	0	0	<0.001
	% within physical activity level per weekday	97.7%	2.3%	0.0%	0.0%	0.0%	
Always	Count	4	13	1	3	0	
	% within physical activity level per weekday	19.0%	61.9%	4.8%	14.3%	0.0%	
Sometimes	Count	70	12	31	5	1	
	% within physical activity level per weekday	58.8%	10.1%	26.1%	4.2%	0.8%	
Rarely	Count	111	118	16	91	3	
	% within physical activity level per weekday	32.7%	34.8%	4.7%	26.8%	0.9%	
Never	Count	1	35	2	2	42	
	% within physical activity level per weekday	1.2%	42.7%	2.4%	2.4%	51.2%	

**Table 6:** Association between time spent on physical activity and the participants’ profession.

Physical Activity per Week		Profession						*p*-Value
		Businessman *n* (%)	Housewife *n* (%)	Students *n* (%)	University Employee *n* (%)	Government Employee *n* (%)	Private Employee *n* (%)	
Often	Count	0	0	127	1	3	1	<0.001
	% within physical activity level per weekday	0.0%	0.0%	96.2%	0.8%	2.3%	0.8%	
Always	Count	0	2	0	17	0	2	
	% within physical activity level per weekday	0.0%	9.5%	0.0%	81.0%	0.0%	9.5%	
Sometimes	Count	20	1	39	22	34	3	
	% within physical activity level per weekday	16.8%	0.8%	32.8%	18.5%	28.6%	2.5%	
Rarely	Count	0	2	156	18	121	42	
	% within physical activity level per weekday	0.0%	0.6%	46.0%	5.3%	35.7%	12.4%	
Never	Count	0	35	1	8	36	2	
	% within physical activity level per weekday	0.0%	42.7%	1.2%	9.8%	43.9%	2.4%	

**Table 7:** Information about the respondents’ attitudes toward sedentary behavior.

Statements	Strongly Agree *n* (%)	Agree *n* (%)	Neutral *n* (%)	Disagree *n* (%)	Strongly Disagree *n* (%)	Mean (Std)
Sitting for long periods can be harmful to health.	467 (67.4)	89 (12.8)	56 (8.1)	77 (11.1)	4 (0.6)	3.32 (1.106)
Sitting for prolonged periods will not negatively impact my health if I regularly exercise during the day	13 (1.9)	209 (30.2)	311 (44.8)	128 (18.5)	32 (4.6)	2.32 (1.067)
Sitting for prolonged periods can negatively impact my Mental and emotional state	132 (19)	444 (64.1)	68 (9.8)	44 (6.3)	5 (0.7)	1.86 (1.244)
There is a strong relationship between backpain and prolonged sitting hours	421 (60.8)	128 (18.5)	95 (13.7)	43 (6.2)	6 (0.9)	3.19 (1.186)
There is a strong relationship between social media and prolonged sitting hours	131 (18.9)	364 (52.5)	108 (15.6)	87 (12.6)	3 (0.4)	2.02 (1.216)
There is a strong relationship between coffee-dessert time and prolonged sitting hours	120 (17.3)	286 (41.3)	177 (25.5)	103 (14.9)	7 (1.0)	2.22 (1.186)

## Data Availability

The data used in this study can be obtained from the corresponding author upon request.
